# Supplementary material for: Comprehensive Evaluation of the Effects of Hot Air Drying Temperature on the Chemical Composition, Flavor Characteristics and Biological Activity of Houttuynia cordata Thunb
Source: Foods. 2025 May 30;14(11):1962. doi: 10.3390/foods14111962 (PMC12154787; doi:10.3390/foods14111962)
Supplement: Supplementary file 1 [file foods-14-01962-s001.zip › foods-3661003-supplementary.pdf]

Supplementary Materials

Table S1. Fitting results of dynamic thin layer drying model of *Houttuynia cordata Thunb.* dried using hot air at different temperatures.

Model	SP	40℃	50℃	60℃	MSP
Lewis/Newton	R^2	0.9986	0.9913	0.9854	0.9918
	$RMSE$	0.0121	0.0315	0.0416	0.0284
	RSS	0.0063	0.0397	0.0639	0.0366
	χ^2	0.0002	0.0010	0.0018	0.0010
Page	R^2	0.9988	0.99648	0.99862	0.9980
	$RMSE$	0.0110	0.0198	0.0126	0.0145
	RSS	0.0052	0.0157	0.0059	0.0089
	χ^2	0.0001	0.0004	0.0002	0.0002
Henderson/Pabis	R^2	0.9987	0.99296	0.99096	0.9942
	$RMSE$	0.0117	0.0280	0.0323	0.0240
	RSS	0.0059	0.0313	0.0385	0.0252
	χ^2	0.0001	0.0008	0.0011	0.0007
Two-term	R^2	0.9986	0.9969	0.9985	0.9980
	$RMSE$	0.0116	0.0181	0.0128	0.0142
	RSS	0.0058	0.0131	0.0061	0.0083
	χ^2	0.0001	0.0004	0.0002	0.0002
Two-term exponential	R^2	0.9989	0.9967	0.9982	0.9979
	$RMSE$	0.0105	0.0193	0.0144	0.0147
	RSS	0.0048	0.0149	0.0077	0.0091
	χ^2	0.0001	0.0004	0.0002	0.0002
Logarithmic	R^2	0.9993	0.9951	0.9939	0.9961
	$RMSE$	0.0082	0.0229	0.0261	0.0191
	RSS	0.0029	0.0211	0.0252	0.0164
	χ^2	0.0001	0.0006	0.0007	0.0005
Midilli	R^2	0.9993	0.9969	0.9988	0.9983
	$RMSE$	0.0084	0.0181	0.0115	0.0127
	RSS	0.0030	0.0131	0.0049	0.0070
	χ^2	0.0001	0.0004	0.0001	0.0002
Aghabashlo	R^2	0.9991	0.9987	0.9989	0.9989
	$RMSE$	0.0096	0.0122	0.0114	0.0111
	RSS	0.0040	0.0060	0.0047	0.0049
	χ^2	0.0001	0.0002	0.0001	0.0001
Wang and Singh	R^2	0.9700	0.9510	0.9588	0.9599
	$RMSE$	0.0555	0.0738	0.0689	0.0661
	RSS	0.1325	0.2179	0.1754	0.1753
	χ^2	0.0032	0.0057	0.0050	0.0047
Silva	R^2	0.9986	0.9913	0.9854	0.9918
	$RMSE$	0.0121	0.0315	0.0421	0.0286
	RSS	0.0063	0.0397	0.9854	0.3438
	χ^2	0.0002	0.0010	0.00183	0.0010
